# Supermarket retailers’ perspectives on healthy food retail strategies: in-depth interviews

**DOI:** 10.1186/s12889-018-5917-4

**Published:** 2018-08-16

**Authors:** Olivia Martinez, Noemi Rodriguez, Allison Mercurio, Marie Bragg, Brian Elbel

**Affiliations:** 1 Department of Population Health, NYU School of Medicine, 227 E 30th St, 6th Floor, Office 626, New York, NY 10016 USA; 2 NYC College of Technology of the City University of New York, New York, 11201 USA

**Keywords:** Healthy food retail, Four Ps, Food environment

## Abstract

**Background:**

Excess calorie consumption and poor diet are major contributors to the obesity epidemic. Food retailers, in particular at supermarkets, are key shapers of the food environment which influences consumers’ diets. This study seeks to understand the decision-making processes of supermarket retailers—including motivators for and barriers to promoting more healthy products—and to catalogue elements of the complex relationships between customers, suppliers, and, supermarket retailers.

**Methods:**

We recruited 20 supermarket retailers from a convenience sample of full service supermarkets and national supermarket chain headquarters serving low- and high-income consumers in urban and non-urban areas of New York. Individuals responsible for making in-store decisions about retail practices engaged in online surveys and semi-structured interviews. We employed thematic analysis to analyze the transcripts.

**Results:**

Supermarket retailers, mostly representing independent stores, perceived customer demand and suppliers’ product availability and deals as key factors influencing their in-store practices around product selection, placement, pricing, and promotion. Unexpectedly, retailers expressed a high level of autonomy when making decisions about food retail strategies. Overall, retailers described a willingness to engage in healthy food retail and a desire for greater support from healthy food retail initiatives.

**Conclusions:**

Understanding retailers’ in-store decision making will allow development of targeted healthy food retail policy approaches and interventions, and provide important insights into how to improve the food environment.

## Background

The obesity epidemic has been a major public health concern over the past decade, with approximately 39.6% of adults and 18.5% of 2–19 year olds now obese in the United States [1]. Calorie consumption is the largest contributor to the obesity epidemic compared to physical inactivity and genetic factors [2], influenced largely by the ubiquity of energy dense and processed foods [3, 4]. Poor diet also contributes to the prevalence of other preventable chronic diseases such as diabetes, hypertension, and heart disease [5, 6]. Food store purchases (i.e., from supermarkets, grocery stores, convenience stores, and specialty food stores) account for 63–76% of total energy intake among Americans [7], with supermarkets being a dominant shopping venue [8].

To increase more healthful food purchases, some supermarkets have begun making their own store-level changes, such as adding healthy checkout lanes devoid of less healthy food products [9]; enhancing the appeal of more healthy foods through colorful packaging, signage, and lighting [10]; offering more healthy food samples; and providing additional nutritional information [10]. Research on supermarkets suggests there are benefits to food choice through in-store product placement, promotion [11–14], and pricing [15–17]; however, it is not clear if such efforts—largely experimental or interventional—are sustainable in healthy food retail. Existing research, mostly examining small food stores, suggests that food retailers are challenged by higher costs, limited demand, and few supplier incentives for more healthy foods [18–20]. Within this body of research, few have sought to uncover food retailers’ perspectives on healthy food retail and their stocking practices [18, 19, 21, 22], and even fewer have used a qualitative methodology to do so [19, 22]. Pinard, et al. (2016) and Kim, et al. (2017) explored food retailers’ perceptions about business practices and factors that encourage or inhibit healthy food retail through in-depth interviews [19, 22]. Their research adds context to small food store retailers’ concerns about profitability, survivability, and competition; pressure from suppliers and manufacturers; consumer demand; and their role in influencing food decisions within their communities. However, like most other food retailer studies in public health, the aforementioned work only sampled small food store retailers [19, 22]. It is not clear how transferrable their findings are to supermarkets, which have a different business structure compared to small food stores.

Using in-depth interviewing to understand the experience of food retailers can provide insight into their business practices and can help identify public health solutions tailored to supermarket retailers to improve food environments and positively influence consumers’ food purchases [22–24]. This is critical given that supermarket retailers—store owners, store managers, and corporate managers—play a central role in shaping the food retail environment. Retailers decide which products are ordered from suppliers, where some products are placed in the store [25], and how appealing signage and shelving look around various types of products [26]. In doing so, supermarkets—a primary source of food with a major influence on the community food environment [27, 28]—have the potential to make impactful changes to the food environment.

The current study employs a qualitative methodology to explore supermarket retailers’ experiences with healthy food retail and to catalogue elements of the complex relationships between supermarket retailers and their customers and suppliers. Based upon the Four Ps framework (i.e., product, placement, promotion, and pricing retail strategies) and two additional factors of personnel and presentation, we examined: 1) retailers’ business practices and 2) retailers’ experiences with healthy food retail, including perceived barriers and motivators.

## Methods

### Design

We employed multiple methods by conducting online surveys and engaging in semi-structured, in-person interviews with a convenience sample of 20 New York State (NYS) supermarket retailers. Supermarkets were defined using the standards set by the New York City Food Retail Expansion to Support Health (FRESH) Program, which characterizes them as full-service stores offering groceries, meat, and produce, and with at least 6000 square feet of sales floor [29]. This is not a standard threshold in other localities, but it is used in New York City where the current study was primarily conducted. To identify differences in implementation of healthy food retail strategies, we sought to recruit retailers in urban and non-urban areas, as well as from low- and high- income neighborhoods (at least two stores were recruited from tracts in the top third of income distribution) [30]. Stores were identified from a list of supermarkets and managers provided to us by the New York City Department of Health and Mental Hygiene (NYC DOHMH), as well as via Google Maps. Stores were classified as urban if they existed within an “urbanized area” (i.e., an area with “50,000 or more people”) as defined by the 2010 United States Census Bureau; stores not matching this definition were classified as non-urban [31]. Store owners or managers were recruited via telephone and screened for eligibility criteria. Participants had to be at least 18 years of age and responsible for making store-level decisions about procurement, placement, and retail. Verbal consent to participate in the study was obtained from all participants over the phone during this initial contact. This study and its procedures were approved by the New York University Institutional Review Board.

We convened a national advisory board of experts (i.e., researchers, health department representatives, and public policy experts who focus on the retail environment) to help develop two data collection instruments for this study—an online survey and an in-person interview guide. Both instruments addressed the Four Ps, as well as two additional factors: personnel and presentation. The online survey included 32 multiple choice questions and was administered via a web link that was sent to participants via email after they provided verbal consent during an initial screening phone call. The online survey captured demographic information on respondents and their stores’ characteristics, as well as their business practices—including factors that influence food product selection, sources of food products, and use of Four Ps strategies for both more and less healthy foods.

Then, a qualitative methodology was employed using 30-min, semi-structured interviews [32]. These interviews were facilitated by one of two project staff members (i.e., a research coordinator and a graduate research assistant) with experience in conducting qualitative research. Discussions took place either in-person or over the phone, in English, and were audiotaped. Open-ended questions with prompts were used to address retailers’ relationships with suppliers; practices related to and sense of control over product selection, placement, and pricing/promotion; perceptions about more healthy versus less healthy products; and experiences with and interest in healthy food retail. Recordings of the interviews were transcribed by a professional transcription service. Each participant was offered a $50 gift card as compensation for his/her time. Data were collected from September to December 2016.

### Analysis

Online survey responses were compiled into frequencies (i.e., we summed participant and store characteristics as well as awareness and use of healthy food retail marketing strategies). The interview transcripts were analyzed using a multistage thematic analysis method [33]. Three trained coders (the research coordinator and two graduate research assistants) individually read each interview transcript to identify themes and develop codes based on emergent themes and interview guide questions [34]. (See Table 1 for the final coding scheme.) Using a multistage thematic analysis approach [35, 36], transcripts were coded through several rounds of individual review and discussion, in which coding discrepancies were discussed until consensus was reached among the three coders. All final coding was completed in ATLAS.ti software (version 7, Berlin, Germany; 2012), and coders subsequently analyzed each quotation by code to identify similarities and differences [35], as well as to quantify the degree of concurrence of key findings across interviews.

**Table 1:** Coding Scheme for Healthy Food Retail: New York City and Upstate New York, 2016

Family	Code Name with Definition
Business Practices	Ordering and stocking practices: descriptions of the processes of ordering and stocking products
	Product placement: how retailers decide where to place products in their stores
	Market Research: whether stores seek out information about product trends, and how they use it
	Pricing: the process of setting product prices and the factors that influence them
	Personnel roles: descriptions of store staff and their responsibilities
Relationship with Food Suppliers	Retailers’ independence: the extent to which retailers retain independence and control of decision-making in their stores
	Influence of supplier: ways in which distributors/suppliers influence retailers to purchase and supply products
Relationship with Customers	Knowing one’s customer base: being familiar with the community’s demographics and preferences
	Empowering customers: ways in which customers are encouraged to engage with retailers
	Perceptions of customer behavior: how grocers believe their customers shop for and relate to food
Healthy Food Retail	Perceptions of healthy foods: what retailers believe constitute healthy food options
	Efforts to engage in healthy retail: what retailers have previously or are currently doing to promote healthy eating
	Barriers to healthy retail: factors that make healthy retail difficult or decrease feasibility
	Motivators for healthy retail: factors that increase the likelihood of engaging in healthy retail
	Willingness to engage in healthy retail: retailers’ future intent to participate in or openness to healthy retail

## Results

Data were analyzed in December 2016. The final sample included 20 retailers, after contacting 112 supermarkets in target areas of NYS (response rate of 18%). Sixteen individuals worked at independent stores (privately owned store or chain of stores); one at a cooperative; one at a non-independent store (affiliated with or owned by another company); and two at national chain headquarters. The majority of retailers (83%, not including those from national chain headquarters who did not represent a specific store location) were located in low-income neighborhoods (i.e., areas from census tracts in the bottom third of income distribution) because obesity rates are highest in these tracts and there is a need for strategies that are sensitive to consumers in these areas [37]. Two-thirds of retailers were located in urban areas (66%, not including those from national chain headquarters who did not represent a specific store location), while one-third of retailers were located in non-urban areas (33%). Participants included supermarket owners (30%), managers (60%), and corporate managers (10%), and more than half reported over 10 years of experience working in the grocery store business (55%). They were primarily male (70%), non-Hispanic white (60%) or Hispanic (35%), and had a mean age of 48 years. The stores participants operated were primarily single location outlets. See Table 2 for further participant and store characteristics.

**Table 2:** Online Survey, Participant and Store Characteristics, New York, 2016

	Total		NYC		Upstate NY		Chain Headquarters	
	n	%/SD	n	%/SD	n	%/SD	n	%/SD
Position/role								
Corporate category manager	1	5%	0	0%	0	0%	1	50%
Corporate nutritionist	1	5%	0	0%	0	0%	1	50%
Owner, multiple stores	2	10%	1	9%	1	14%	0	0%
Store manager/area manager	12	60%	8	73%	4	57%	0	0%
Store owner	4	20%	2	18%	2	29%	0	0%
Type of business								
Independent – store or chain of stores is privately owned	15	75%	9	82%	6	86%	0	0%
Not independent – store is affiliated with or owned by another company	1	5%	1	9%	0	0%	0	0%
Corporate headquarters for a large chain store	2	0%	0	0%	0	0%	2	100%
Co-operative	1	5%	0	0%	1	14%	0	0%
Other	1	5%	1	9%	0	0%	0	0%
Number of store locations								
Just this location	11	55%	5	45%	6	86%	0	0%
2–5 locations	6	30%	5	45%	1	14%	0	0%
More than 10 locations	1	5%	1	9%	0	0%	0	0%
51–100	1	5%	0	0%	0	0%	1	50%
More than 100 locations	1	5%	0	0%	0	0%	1	50%
Length of employment								
1–5 years	6	30%	4	36%	1	14%	1	50%
5–10 years	3	15%	3	27%	0	0%	0	0%
More than 10 years	11	55%	4	36%	6	86%	1	50%
Years in grocery store business								
1–5 years	1	5%	1	9%	0	0%	0	0%
5–10 years	2	10%	2	18%	0	0%	0	0%
More than 10 years	17	85%	8	73%	7	100%	2	100%
Gender								
Female	6	30%	1	9%	4	57%	1	50%
Male	14	70%	10	91%	3	43%	1	50%
Race/ethnicity								
Hispanic	7	35%	7	64%	0	0%	0	0%
Black, African American	2	10%	2	18%	0	0%	0	0%
White	12	60%	3	27%	7	100%	2	100%
Other race or multi-racial:	6	30%	6	55%	0	0%	0	0%
Mean [SD] Age	47.7	[13.48]	42.4	[14.69]	52.0	[8.76]	61.5	[3.54]
Approach to distribution								
We rely on a distributor that is a completely separate company	15	75%	9	82%	6	86%	0	0%
We handle our own distribution	1	5%	1	9%	0	0%	0	0%
Other:	4	20%	1	9%	1	14%	2	100%
Mean store square feet (*1000)^a^	24.7	[30.73]	19.4	[20.65]	16.9	[32.42]	76.0	[19.80]
Mean proportion [SD] of customers from the following groups:								
African American	31.5%	[25.32]	44.4%	[22.99]	13.0%	[15.49]	N/A	
Hispanic	18.7%	[18.71]	29.5%	[13.99]	3.3%	[3.30]	N/A	
Asian	3.7%	[3.79]	3.2%	[4.29]	4.3%	[3.14]	N/A	
White	41.5%	[37.36]	14.3%	[17.48]	76.4%	[23.47]	N/A	
Seniors	19.6%	[12.23]	15.6%	[10.75]	25.3%	[12.70]	N/A	
Youth	20.7%	[18.31]	21.2%	[21.03]	20.0%	[15.17]	N/A	
Families	45.8%	[25.66]	48.7%	[24.59]	41.6%	[28.54]	N/A	
SNAP recipients	42.5%	[31.51]	60.0%	[26.61]	22.4%	[24.75]	N/A	
WIC recipients	29.9%	[26.32]	34.2%	[29.36]	17.0%	[9.90]	N/A	
Accepted forms of payment								
Cash	20	100%	11	100%	7	100%	2	100%
Credit cards	19	95%	10	91%	7	100%	2	100%
Debit cards	20	100%	11	100%	7	100%	2	100%
Checks	9	45%	2	18%	5	71%	2	100%
EBT (SNAP/food stamps)	20	100%	11	100%	7	100%	2	100%
WIC	12	60%	8	73%	2	29%	2	100%
Store credit	8	40%	6	55%	2	29%	0	0%
ApplePay/Android Pay/Google Wallet	7	35%	4	36%	1	14%	2	100%
None	1	5%	1	9%	0	0%	0	0%
Gift Cards	2	10%	1	9%	1	14%	0	0%
Participate in NYC DOHMH healthy retail programs								
Shop Healthy NYC	1	5%	1	9%	0	0%	0	0%
FRESH	2	10%	2	18%	0	0%	0	0%
Other:	3	15%	1	9%	2	29%	0	0%
N/A	1	5%	0	0%	0	0%	1	50%
None	12	60%	7	64%	5	71%	0	0%
Don’t know	1	5%	0	0%	0	0%	1	50%
Sell locally produced foods								
Yes	16	80%	7	64%	7	100%	2	100%
No	2	10%	2	18%	0	0%	0	0%
Not sure	2	10%	2	18%	0	0%	0	0%
If so, which locally produced foods offered								
Dairy	13	65%	6	55%	6	86%	1	50%
Fruits	17	85%	9	82%	6	86%	2	100%
Vegetables	16	80%	7	64%	7	100%	2	100%
Meat/fish	12	60%	5	45%	5	71%	2	100%
Other:	4	20%	2	18%	1	14%	1	50%
Sources of food products								
Food wholesaler	12	60%	5	45%	6	86%	1	50%
Food distribution center	11	55%	7	64%	3	43%	1	50%
Warehouse club	3	15%	1	9%	2	29%	0	0%
A middle man or independent delivery person	9	45%	6	55%	2	29%	1	50%
Directly from a food supplier or manufacturer	16	80%	8	73%	6	86%	2	100%
Small food producer (including farmers and small specialty businesses)	12	60%	4	36%	7	100%	1	50%
Factors influencing food product selection, means [SD]^b^								
Consumer demand	4.7	[0.99]	4.6	[1.21]	4.7	[0.76]	5.0	0
Owner or store HQ suggestion	3.6	[1.43]	3.5	[1.57]	3.7	[1.37]	4.0	[1.41]
Written agreement or contract with supplier	2.2	[1.33]	1.8	[1.32]	2.8	[1.48]	2.5	[0.71]
Supplier discount	3.4	[1.23]	3.2	[1.47]	3.9	[0.69]	2.5	[0.71]
Volume of sales or profit margin	4.1	[1.24]	4.3	[1.19]	3.7	[1.51]	4.5	[0.71]
Industry data	3.8	[1.11]	4.1	[0.93]	3.1	[1.22]	4.5	[0.71]
Whether supplier will stock the product	1.8	[1.37]	1.6	[1.33]	2.5	[1.73]	1.5	[0.71]
Whether supplier will create a planogram for the product category	1.5	[0.82]	1.7	[1.12]	1.3	[0.50]	1.0	0
Available shelf space	3.2	[1.50]	3.4	[1.65]	3.4	[0.98]	1.0	0
Product category	3.2	[1.23]	3.4	[1.51]	2.7	[0.76]	4.0	0
Product brand	3.2	[1.20]	3.1	[1.54]	3.0	[0.82]	4.0	0
Displays, signs, or shelf tags provided by supplier	1.8	[1.07]	2.1	[1.46]	1.6	[0.53]	1.5	[0.71]
Promotion in a store circular, on store website, store radio, or local media (cooperative advertising)	3.6	[1.39]	3.7	[1.56]	3.9	[1.07]	2.0	0
Packaging	2.8	[1.20]	3.2	[1.20]	2.6	[1.27]	2.0	0
“Very important” to offer the following:								
Quality of food	19	95%	10	91%	7	100%	2	100%
Availability of food (variety, brands)	17	85%	9	82%	6	86%	2	100%
Competitive prices	15	75%	8	73%	5	71%	2	100%
Good customer service	18	90%	9	82%	7	100%	2	100%
Convenient business hours	18	90%	10	91%	6	86%	2	100%
Store cleanliness	19	95%	10	91%	7	100%	2	100%
N	20		11		7		2	

^a^Note: Square footage measures, where indicated, were obtained from the New York State Office of Information Technology Services

^b^Note: Responses were collected on a scale of 1 to 5 where 1 = least influential and 5 = most influential

The ensuing key qualitative findings are based on the degree of concurrences across interviews related to retailers’ business practices and their experiences with healthy food retail efforts. From this emerged common retail practices within the Four Ps framework and more healthy food retail efforts experienced by the sampled supermarket retailers, such that the results are framed around these key themes. Within the context of each theme, the results highlight factors that either supported retailers’ business practices or factors that challenged their efforts to engage in more healthy food retail.

### Retailers’ business practices

#### Product selection

Both online survey and qualitative results suggest that managers believe customer demand is the most important factor with regard to product selection (See Table 2). One retailer summarized the importance of the customer when stating:

“So the customers are really our main, key players. We’re here for them so we have to know exactly what they want, and making a decision is really so much about what the customer really wants […]” (Urban, High Income, Interview 11).

Nearly all retailers noted that they select products from multiple food suppliers to accommodate special requests from customers and to obtain a wider variety of products: “…a good portion of our stuff that we do is on customer suggestions…” (Non-urban, Low Income, Interview 6).

Consequently, many retailers identified the lack of customer demand for more healthy foods as a barrier to the success of healthy retail efforts: “People tend to go more for what’s unhealthy” (Urban, Low Income, Interview 1). As explanation, a few retailers mentioned the negative impact of manufacturers’ pervasive marketing of less healthy food on customer demand pushing them to carry these foods.

“…if the manufacturers create the demand, then we are obliged to carry it. Or as if they did advertising, they succeed in making everybody aware – to buy something that we have not carried, we can be obliged to carry it.” (Urban, High Income, Interview 9)

Also influential to product selection are supplier suggestions and incentives, which include discounts on bulk orders, buy one get one half off promotions, and introductory deals for trying new products (e.g., free product, lower initial prices, or reimbursement for un-purchased product).

“We used to be exclusively Pepsi providers and Coca-Cola has always been right at the door pushing and offering rebates and incentives and discounts. And we did cave this last year, so now we offer both.” (Non-Urban, Low Income, Interview 4)

Although discounts—highly influential for product selection—were often offered for less healthy foods, a few retailers in urban, low-income areas noted that vendors had tried to promote more healthy foods. But once familiar with the retailer’s catchment area and its demand, the vendors became cautious about providing such items:

*Interviewer*: “That’s interesting. Vendors have tried to get you to stock healthier stuff?”

*Interviewe*e: “You know what it is? If the vendors already have a couple years around this zone, they know, also, what to push and what not to push.” (Urban, Low Income, Interview 2)

Retailers in non-urban areas also discussed having fewer vendors to work with as a limitation to product selection.

“I think our big - our bigger problem than that is just a lack overall of more distributors. You know, we just lack - we just lack - a variety of distributors. We lack a competition of distributors - in this area, whereas downstate and across the lake in Vermont, they have access more to New England markets […]” (Non-Urban, Low Income, Interview 1)

“[…] A lot of companies just don’t have the personnel to send up this far. And being that I’m just a little independent, one independent store, they pretty much concentrate on the chain stores.” (Non-Urban, Low Income, Interview 3)

Another limitation some retailers discussed was being required to adhere to corporate lists of approved suppliers:

“I can order from specific pre-approved vendors. Now if any new vendors coming in, they have to go to corporate office and to be cleared.” (Urban, Low Income, Interview 8)

Other retailers similarly said they are subject to store policies for the types of products purchased (e.g., list of ingredients or genetically modified organism (GMO) items that must be avoided):

“[…] there are some restrictions. We have a list of additives that we try to avoid.” (Urban, High Income, Interview 10)

Additionally, more than half of retailers said they subscribe to some type of supermarket magazine or newsletter for product suggestions, and nearly half said they assess what their competitors offer.

“Occasionally, I go to a bigger store […] we'll just look around, check out something, or check out prices, check out products, see what they have that's new, what they seem to be stocking a lot of, and try to get some ideas from that as well.” (Non-Urban, Low Income, Interview 3)

Despite a variety of factors, some perceived as limitations, influencing product selection, retailers expressed having the final say on the content of initial orders and subsequent restocking for their stores.

"Now, if they [suppliers] really really push something, then, they might influence us a little more, but ultimately we have all the say. There's nothing we have to have in the store. If we don't want it we don't take it." (Urban, Low Income, Interview 3)

“If we have a farmer that has a crop of, let’s say, blueberries that he’s looking to sell at our locations, we would taste them and sample them and obviously get the best price. But they’re all our decisions.” (Non-Urban, Low Income, Interview 4)

However, exceptions were noted most commonly for direct store delivery products, such as certain bread, beverage, snack, and gourmet items, which are often ordered and stocked by company representatives, not in-store staff [38, 39].

“For the most part, we stock it all ourselves. The only time that we don’t stock it is the breads, meat, Entenmann’s cake. They do the stocking and they put it in the merchandise.” (Urban, Low Income, Interview 1)

While discussing product selection, retailers were asked about their stores’ more and less healthy food mix. Most retailers reported that they offer a greater amount of less healthy than more healthy food options, largely driven by customer demand and supplier offerings. Interestingly, retailers shared differing ideas about how they define healthy foods. Most mentioned produce, or fruits and vegetables; however, there was less agreement on other categories to include. Some considered unprocessed, whole grain, low sugar, low sodium, and low fat foods to be healthy, while others focused on labels like organic, all natural, grass-fed, and local.

#### Product placement

Product placement factors commonly discussed were supplier suggestions, market changes, and product visibility enhancements. Supplier suggestions via planograms (defined as a plan for displaying merchandise in a store in a way that maximizes sales [40]) were noted as being somewhat influential at least initially during the store planning phase. However, retailers acknowledged the importance of maintaining a flexible in-store design to adapt to market changes (e.g., product lines closing or new ones appearing). Retailers discussed not being bound by contract or another agreement to permanently adhere to a given plan and having the authority to accept or modify a planogram as desired.

“Previously, when we first opened our supplier came in and had some basic planograms to get us started. But, over the time, we have definitely moved stuff where it's made more sense [...]” (Non-Urban, Low Income, Interview 5)

Product visibility was another important consideration for product placement, primarily for its ability to influence customer behavior. For example, retailers noted that changing product locations would encourage customers to explore the store and notice new or featured products, thereby increasing sales.

“You always have to evolve and move things here and there. People get tired of seeing the same thing too. They like to see change. You’ll see the difference in sales –just by resetting something. Maybe shift things around a little bit, evolving it –that will boost sales.” (Urban, Low Income, Interview 3)

Online survey responses showed that the most commonly used healthy food placement strategies included placing fruits and vegetables near the front of the store or by an entrance, at eye level, or on end caps. These findings are also evident in the interviews.

“People here don’t know what Quinoa is […] but if you place it in the front, it’s like a grab and go cup you put a decent price on it, one person might try it. I did this three weeks ago. We ordered five cases. We already had to do another order, and a customer came to me and wants to buy a whole case. The placement of the product is real important. There are certain areas where a customer, no matter what, will see it and be more enticed to buy it.” (Urban, Low Income, Interview 3)

“I think they’re shifting away from unhealthier items because as we move some of our healthier items to our main line aisles, the higher traffic aisles, they’re having a lot of success.” (National Chain, Interview 2)

According to the online surveys, most retailers have tried using at least one placement strategy to sell healthy foods (see Table 3).

**Table 3:** Online survey, Awareness and Use of Healthy Food Retail Marketing Strategies, New York, 2016

	Total		NYC		Upstate NY		Chain Headquarters	
	n	%	n	%	n	%	n	%
Health promotion signage								
Food Guide Pyramid or My Pyramid	1	5%	0	0%	0	0%	1	50%
5-A-Day	2	10%	0	0%	1	14%	1	50%
Fruits and Veggies - More Matters	2	10%	0	0%	2	29%	0	0%
Rethink Your Drink	0	0%	0	0%	0	0%	0	0%
Nutritional information	6	30%	1	9%	3	43%	2	100%
Healthy recipes	4	20%	0	0%	2	29%	2	100%
Locally grown produce (i.e. grown in New York or New Jersey)	7	35%	0	0%	6	86%	1	50%
None of the above	11	55%	10	91%	1	14%	0	0%
Other	1	5%	0	0%	1	14%	0	0%
Use of healthy placement strategies								
*Healthy checkout aisles*								
Have used	5	25%	2	18%	1	14%	2	100%
Willing to try	3	15%	2	18%	1	14%	0	0%
*Healthy coolers or vending machines*								
Have used	6	30%	2	18%	2	29%	2	100%
Willing to try	3	15%	1	9%	2	29%	0	0%
*Placing fruits and vegetables near front of store or by entrance*								
Have used	16	80%	8	73%	6	86%	2	100%
Willing to try	2	10%	2	18%	0	0%	0	0%
*Placing healthy foods at eye level*								
Have used	16	80%	8	73%	7	100%	1	50%
Willing to try	3	15%	2	18%	0	0%	1	50%
*Placing healthy foods on end caps*								
Have used	12	60%	6	55%	4	57%	2	100%
Willing to try	4	20%	3	27%	1	14%	0	0%
*Other secondary placements of healthy foods (by deli, in bakery,* etc.*)*								
Have used	9	45%	4	36%	3	43%	2	100%
Willing to try	5	25%	3	27%	2	29%	0	0%
*Candy free zones*								
Have used	2	10%	0	0%	1	14%	1	50%
Willing to try	2	10%	1	9%	1	14%	0	0%
Use of pricing and promotion strategies								
*Buy one get one free*								
Have used	14	70%	8	73%	5	71%	1	50%
Willing to try	0	0%	0	0%	0	0%	0	0%
*Coupons in circulars*								
Have used	12	60%	7	64%	4	57%	1	50%
Willing to try	1	5%	1	9%	0	0%	0	0%
*Coupons online*								
Have used	8	40%	4	36%	2	29%	2	100%
Willing to try	3	15%	2	18%	1	14%	0	0%
*Coupons at the register*								
Have used	11	55%	6	55%	4	57%	1	50%
Willing to try	1	5%	1	9%	0	0%	0	0%
*Store loyalty cards or programs*								
Have used	9	45%	5	45%	2	29%	2	100%
Willing to try	2	10%	2	18%	0	0%	0	0%
*Sales or discounted prices*								
Have used	18	90%	9	82%	7	100%	2	100%
Willing to try	1	5%	1	9%	0	0%	0	0%
*Placing items in high traffic areas or at eye level*								
Have used	16	80%	8	73%	6	86%	2	100%
Willing to try	0	0%	0	0%	0	0%	0	0%
*Signs promoting fruits and vegetables*								
Have used	16	80%	7	64%	7	100%	2	100%
Willing to try	1	5%	1	9%	0	0%	0	0%
*Signs promoting healthy low and no calorie beverages*								
Have used	11	55%	6	55%	3	43%	2	100%
Willing to try	4	20%	2	18%	2	29%	0	0%
*Signs promoting unhealthy foods and beverages*								
Have used	9	45%	4	36%	4	57%	1	50%
Willing to try	1	5%	1	9%	0	0%	0	0%
*Using product tags to advertise healthier options*								
Have used	8	40%	4	36%	2	29%	2	100%
Willing to try	6	30%	2	18%	4	57%	0	0%
*Displaying healthy items from different categories together (*e.g.*, whole grain bread and prepared salad)*								
Have used	10	50%	6	55%	2	29%	2	100%
Willing to try	6	30%	2	18%	4	57%	0	0%
*Front of package nutrition information*								
Have used	1	5%	0	0%	0	0%	1	50%
Willing to try	9	45%	5	45%	4	57%	0	0%
*Kid-oriented characters on produce or healthy items*								
Have used	4	20%	2	18%	0	0%	2	100%
Willing to try	9	45%	3	27%	6	86%	0	0%
*Freestanding displays promoting fruits, vegetables, or whole grains*								
Have used	13	65%	7	64%	4	57%	2	100%
Willing to try	4	20%	1	9%	3	43%	0	0%
N	20		11		7		2	

#### Product pricing and promotion

Online survey responses showed that retailers are less willing to try using pricing strategies than placement strategies to sell healthy foods. However, nearly all retailers reported trying sales or discounted prices, with the most popular being buy one get one free and coupons in store flyers (see Table 3). Interview discussions revealed that the cost of items from the supplier, or availability of special deals or sales for bulk purchases, often determine retailers’ ability to lower in-store prices while maintaining their profit margins.

“[…] we don’t really give discounts on health foods because for the most part it’s more expensive. For the most part, coming from the vendors is more expensive, so we usually do have to put it at a higher price because we try to manage everything at a 30 percent margin, 30 to 35 percent margin. So, basically, we base the sale price on that. Unless we get a deal [...].” (Urban, Low Income, Interview 1)

Some retailers attempted to negotiate with vendors for lower purchase prices, with varying results. Many noted the low frequency of incentives (e.g., discounts) from suppliers on healthy foods. One urban retailer made this point clear, offering his own explanation:

“They don't have too many crazy deals with healthy stuff. For them, it's a higher cost, so they're not going to give it away unless it's really something they really, really want to start pushing.” (Urban, Low Income, Interview 3)

The price of more healthy foods was noted as a major barrier to customer choice in low-income areas. As one retailer pointed out: “But the price ultimately, in many cases, is the determining factor because of people’s budgets” (Urban, Low Income, Interview 4). As well, most retailers noted that items they choose to put on sale are often based on customer demand: “[…]it’s really what we would perceive the consumer interest would be in it and whether it’s something that a person would buy extra of just because it’s on sale” (Non-Urban, Low Income, Interview 1). Yet even with price reductions, healthy items were not guaranteed to sell: “[…] you can reduce the price, but a lot of times it’s just a lack of demand for the particular product that’s why it’s not moving” (Non-Urban, Low Income, Interview 3).

It should be noted that, as with product selection, many retailers are dependent on a list of predefined sales authorized by their main supplier/wholesaler and have limited ability to further shift prices without operating at a loss.

“The wholesaler, they send me weekly the weekly sales. In other words, this is what I put and this is the weekly sale book that they send me. What I do is I pick what would sell in my zone.” (Urban, Low Income, Interview 2)

Key promotion strategies for more healthy foods were signage, in-store demos, and product samples. One non-urban retailer noted: “And certainly demos, having them try stuff is important” (Non-Urban, Low Income, Interview 5). Two urban retailers added:

“[…] we have a deal on cantaloupe, we cut one up, we put it by the front. Those that might not pick it up say, "You know, it's free. Let me taste it. You know what, let me take a cantaloupe." […] Those sampling helps.” (Urban, Low Income, Interview 6)

“[…] we do a weekly special. We do flyers. We send it out by mail and it’s also available on our website to show the product of the week. […] we also request a lot of demo by the company when they come down here. They’re educating customers and they educate us, as well.” (Urban, High Income, Interview 11)

Whether it was product selection, placement, pricing, or promotion, some retailers perceived their relationship with suppliers as a partnership such that working together would benefit all parties, including the customer.

“They are our partners so we have to work together. So we are in the same industry. They supply us and we sell it. We’re pretty much the middlemen between them and the customers.” (Urban, High Income, Interview 11)

“I always tell everybody with vendors it's a relationship – us working together also helps the customer too. It works for me, works for you; I sell it to the customer who gets a good price.” (Urban, Low Income, Interview 3)

Even while promoting partnerships with food industry stakeholders and at times recognizing the constraints under which business practice decisions are made, retailers emphasized their control over final decisions:

“…at the end of the day, it’s what I say goes because – it’s my business. I’m paying for every square feet, I’m paying rent” (Urban, Low Income, Interview 2).

Interestingly, one retailer described a perceived difference between chain and independent supermarket operations, one that may provide independents with more decision-making freedom.

“Perhaps the larger chains they already have a fixed system of how they do things and they’re a little more systematic and they probably have to listen more to what gets done at headquarters, but we do have a little more freedom in that sense. Sometimes that may even be beneficial because we can make a quicker decision in adapting to something.” (Urban, Low Income, Interview 4)

### Experience with healthy food retail

All retailers discussed having used at least one product placement, pricing, or promotion strategy to engage in healthy food retail. However, most NYC retailers—particularly those in low-income areas—did not believe their healthy food retail efforts made a difference: “I’ve been personally trying to introduce organics and specialties, but when there isn’t that demand, I have to take it back, to pull back a bit” (Urban, Low Income, Interview 4). Another retailer concurred:

“[…] we’ve been working to lean into try to bring in more healthy food. The only issue is that in this community it’s kinda tough because people really don’t go – don’t really – so most of the time we do try to bring it in. It’ll go bad or expires.” (Urban, Low Income, Interview 1)

Whereas, more than half of the retailers located outside of NYC, although in low-income areas, were more optimistic, noting more positive experiences:

“And they came up to me and they wanted Vegenaise mayonnaise […] And so, we give it a shot [...] And it’s actually selling, you know.” (Non-Urban, Low Income, Interview 6)

“I actually work with local farmers and stuff […] to make up for the lack of diversity in the produce section […] people like that because they feel like it's more wholesome than the mass produced stuff that they get.” (Non-Urban, Low Income, Interview 7)

One non-urban retailer noted in regards to getting customers to eat healthy:

“I think that people generally don’t go looking to change their lifestyle that way. So, they need a little bit of a push. So if we can make that information available to them, it might cause them [to change]…” (Non-Urban, Low Income, Interview 5).

Despite some retailers’ optimism and success with more healthy food retail, both non-urban and urban (NYC) retailers in low-income areas perceived their customer base as possessing unhealthy eating habits, which is why they stock less healthy products. One retailer concluded: “Not too many people know about eating healthy […]” (Urban, Low Income, Interview 3). Another’s perspective was: “You would see a shopping cart full of sodas and chips and things are not healthy, that we know is not healthy, but at the end of the day is what people buy” (Urban, Low Income, Interview 1). A third retailer explained:

“I think the challenge is we live in a society that wants instant gratification, and I think many, many people are just lazy. So what’s easier to pick up a bag of chips or to peel an orange? It’s easier to pop open the bag of chips, and that’s not a good, healthy eating choice.” (Non-Urban, Low Income, Interview 4)

On the other hand, retailers in high-income areas believed their customers already had fairly healthy eating habits and could afford to pay for more expensive healthy food items. Consequently, these retailers expressed a greater desire to offer and expand their healthy food retail efforts: “Well, we already have a very large produce section […] our members are always demanding those things” (Urban, High Income, Interview 10).

Despite perceptions of customers’ eating habits, some retailers expressed a personal desire to make their neighborhoods healthier: “I want to have a healthy neighborhood around me, and I want to have healthy people around here” (Urban, High Income, Interview 11). As well, some described the importance of remaining ahead of competitors’ when discussing engagement in healthy food retail:

“One thing we're trying to do here is introduce healthier stuff. We have the best variety in this area, so we want to be the first to introduce this […].” (Urban, Low Income, Interview 3)

Generally, retailers shared their thoughts on what they observed to be a positive cultural shift towards healthy eating, largely driven by health concerns and increased marketing of healthy foods.

"I think a lot of that right now has to do with our culture is changing. I think a lot of people are eating better for sure." (Non-Urban, Low Income, Interview 6)

“We are mindful of the public’s desire for healthy foods...We want to be a reflection of what society is asking for...In other words, we’ve moved away from – the so-called “unhealthy” foods. But the marketplace has also done that as well because the products that are deemed unhealthy have really lost a lot of their sales.” (Urban, High Income, Interview 9)

Along with a change in consumer demand, business opportunities have arisen in the sale of healthy foods. One of the corporate managers explained:

“[…] we also do have a responsibility to provide the best and the healthiest foods that we can. And we do it for selfish reasons; don’t make any mistake about it. We’re doing it because that’s where the growth is, that’s where the profits are, that’s what the consumer wants. […] now you see everybody jumping in and making sure that they have their own natural, organic, edgier products and so on […] And a lot of it’s because of price […] all retail entities competing in the health segment and it’s driving costs down.” (National Chain, Interview 2)

An urban retailer echoed the business perspective, stating:

“[…] even though we want everyone to eat more healthily, it is a business. So whatever is trending is kind of like what they’re going to push. Right now, healthier living is trending.” (Urban, Low Income, Interview 5)

Despite the observed increase in supply and sales of more healthy food, retailers recognized that it will take time before most people prefer to purchase more healthy foods: “that change is going to take time for the simple reason that not everybody believe on the health” (Urban, High Income, Interview 11). Meanwhile, some retailers, particularly those in low-income areas, felt that there is limited progress they can achieve on their own without support from suppliers or the larger food industry, which still often incentivizes less healthy food items: “We try and get them to be - the prices to be in line with what we consider the regular stuff because it doesn’t seem fair that it’s more expensive just because it’s healthier” (Non-Urban, Low Income, Interview 7). Lack of store capacity to develop promotional materials and signage to market more healthy foods in the absence of external support was also a barrier.

“Probably if supplier just offered more of those things for us instead of us having to take the time to do it. Like if in an item comes in from a supplier, if it already comes in with signage and stuff to come up like the pesto did, that's really helpful because we just don't have enough hours in the day, nor do we have the staff to maintain such a thing.” (Non-Urban, Low Income, Interview 7)

Additionally, the same retailer pointed out the disparity in access to resources between independent and chain supermarkets.

*Interviewee*: “I know the bigger chain stores already offer [demos], and they have the personnel and the money to do that kind of stuff. But we don’t.” (Non-Urban, Low Income, Interview 7)

Other support could come from healthy food retail programs, which many retailers had heard of and reported a willingness to participate in, but most mentioned not having been approached by one. Particularly for those retailers in non-urban areas, access to healthy retail programs was said to be limited or completely unavailable.

*Interviewer:* “Thinking about what we would call healthy retail programming, at least here in New York City there are a lot of official city-level programs in place that retailers can use to get some of that signage, to get some help with promoting healthy items. Is that something that is available or exists in your area, or something that you ever tried...”

*Interviewee:* “Not that I know of, no. I don’t know of anything like that. That would be awesome.” (Non-Urban, Low Income, Interview 7)

Therefore, if resources from healthy retail programs could be provided or disseminated more widely, retailers reported that would be a motivator for them to engage in healthy retail and that they would be more likely to utilize those resources.

*Interviewer:* “It seems like you have a positive view over those [healthy retail] programs […] how do you feel about programs like that that try to go into stores and suggest new ways to try promoting products that are healthier, perhaps?”

*Interviewee:* “I think that’s a great idea. We would be open to it. I don’t know how other stores respond but I'm always open to that kind of stuff, especially if there’s help available because that just helps time-wise for us. We’d be willing to do it; we just need somebody that that’s what they do is go around and promote that. If they just sent me signage, no problem putting it up.” (Non-Urban, Low Income, Interview 7)

## Discussion

Using online surveys and in-depth interviews with supermarket retailers in NYS, this study examined supermarket retailers’ perceptions of healthy food retail, the factors that influence their store-level decision-making processes, and their complex relationships within the food industry. Consistent with previous research on healthy food retail (predominantly with small grocers^22^), our findings indicate that supermarket retailers perceive customer demand and suppliers’ product availability and deals as key factors influencing their in-store practices around product selection, placement, pricing, and promotion [18, 20–22]. Only two recent studies focusing on small stores have examined the impact of retailer and distributor relations. The first study by Pinard, et al. (2016) focused on small grocers in rural areas and found that agreements with distributors were particularly influential given competition with chain supermarkets and financial constraints [19]. The second study by Ayala, et al. (2017) concluded that distributors of less healthy products are more likely to promote and obtain stocking agreements for their items through the use of incentives [20]. However, Ayala and colleagues’ methodology did not examine how retailers themselves engaged in the decision-making process.

We add to previous research a unique and rarely seen view, from the supermarket retailer’s perspective, on the utility and impact of in-store healthy food retail strategies. We found that, although suppliers were perceived to influence retail decisions, all retailers still believed they maintain ultimate decision making authority over their business practices. This finding was unexpected in light of strategies such as slotting fees (a way in which food manufacturers obtain premium placement in stores, by paying fees in return for strategic placement of their products; less common today) [25, 41, 42] and other incentives, like discounts for new items, to get new products on retailers’ shelves [43]. Thus, it is unclear how much perception of autonomy matches reality and how this varies by type of supermarket—chain versus independent. Future research should seek perspectives from suppliers and distributors to better elucidate the nature of negotiations that occur between retailers and manufacturers/suppliers and aid in development of more effective healthy food retail strategies.

Furthermore, our analysis reveals insights into the balance between business viability, and the perceived high price and limited demand for more healthy foods which our supermarket retailers identified as a barrier to uptake of healthy food retail strategies (and that is corroborated by other work [18, 19, 21, 22]). Promisingly, industry literature indicates a national trend toward an increased demand for more healthy and fresh food products, such that packaged foods are now competing with fresh foods for shelf space [44, 45]. The comparative costs of more and less healthy foods are likely more nuanced than some retailers first realize. Though it is true that much work has shown that healthier food options often come at a higher monetary cost [46], recent research suggests that some less healthy snacks (e.g., chips) can carry nearly the same price tag as fruits in small food retail settings [47, 48]. A US Department of Agriculture study notes that, when comparing the prices of healthy and unhealthy foods by cost per weight unit or cost per portion size (rather than cost per unit), more healthy foods are nearly equal to or less expensive than their unhealthy counterparts [49]. Healthy food retail programs can intervene through education on the affordability of more healthy foods; by promoting the most recent, accurate cost information to supermarket managers; or by developing economic supports for supermarkets (which have been tested with success in small store settings, as with the Baltimore Healthy Stores Initiative which provided “wholesaler gift cards” to promote stocking of more healthy foods [50]).

Another possible area of focus for healthy food retail programs is retailers’ knowledge and awareness of more healthy foods [51]. Currently, more healthy food retail initiatives tend to target customer behavior [26], given that food choice is personal [52–54]. Yet, retailers are the ones who shape the in-store environment within which the customer makes food purchase decisions. From our interviews, it is apparent that retailer knowledge regarding more healthy foods is not consistent. When retailers were asked to define healthy foods, some focused on perceived unhealthy ingredients such as fat, sugar, and salt; while others focused on perceived healthy descriptions such as organic, natural, local, and grass-fed. These findings support the need to develop interventions to help retailers better understand more healthy food options and be able to translate this knowledge to their in-store practices, a suggestion supported by previous qualitative work with retailers [19]. Empowering retailers in this way can be coupled with in-store promotional activities (e.g., signage, displays, demos) and “choice architecture” (i.e., environmental layout such as product visibility used to influence customers’ food decisions) [26, 55] to maximize impact.

Finally, we point out that healthy food retail remains challenging in the current food marketing environment, where less healthy foods are strongly promoted [56]. Even the most concerted efforts to promote healthy food retail practices may be overwhelmed by the pervasiveness of unhealthy messaging within and outside of stores [57]. Retailers in low-income areas were more likely than those in high-income areas to express not having the financial and staffing resources to combat the heavy promotion of less healthy foods. Healthy food retail is especially challenging in areas that are both low-income and non-urban, where in addition to constrained store resources retailers described experiencing limited access to any healthy food retail initiatives, a finding supported by recent research examining small rural grocery stores [19]. Thus, another important role for more healthy food retail programs should be to target resources to retailers in these areas. In addition to supporting retailers’ healthy efforts, public health interventions should also aim to reduce unhealthy food marketing and unhealthy store-level messaging/signage.

Overall, the results highlight that supermarket retailers work under highly influential factors of supply, demand, and price. Consequently, healthy food retail intervention efforts must be developed and conducted in a way that is mindful of this context. Further, this study highlights the gaps that still exist in retailer access to more healthy food retail support and the need for expansion of healthy food retail programming and other related interventions to improve the food retail environment.

### Limitations

This study is not without limitations. Selection bias is inherent in the methodology used to identify and recruit stores, which were selected from a convenience sample rather than through systematic sampling. Further, our sample includes a high proportion of independent stores, which limits the generalizability of our findings to other types of supermarkets and food retail outlets. A 2011 study done by the Community Development Financial Institutions Fund (CDFI) revealed that chain stores (more than ten locations under one management structure) make up nearly two-thirds of the market for food retail, whereas our sample was 15% chain stores based upon the CDFI’s definition [58]. A greater representation of independent stores (privately owned store or chain of stores [59]) is highly pertinent to our focus on lower-income neighborhoods, where large-format stores like supermarkets and chain stores are less likely to locate [60]. Moreover, independent stores have greater flexibility to make in-store changes than chain supermarkets [61, 62], making them ideally suited for the topic of our interviews. The low response rate (18%) suggests a possible self-selection bias, in that the managers who agreed to participate were perhaps most likely to be health conscious and already interested in healthy food retail. Additionally, although we encouraged participants to be expressive of all of their views, positive and negative, about healthy food retail, we cannot ignore the possibility of a social desirability bias in response to questions about promoting healthy eating. Further, we note that this was a single interview in which retailers seemed to limit the discussion of their financial relationships with suppliers, and of their profit margins and volume. Longer-term relationships may be necessary for these kinds of disclosures. Finally, we would point out that all of our results report perceptions of the retailers we interviewed which may or may not be validated by other sources of objective data.

## Conclusions

Encouraging healthy food retail as a means of improving healthy food access, especially in underserved neighborhoods, is an area of increasing focus nationwide. The retailer’s perspective in this dynamic is critical, yet poorly understood. Results from this study show the complexity of relationships—in particular with customers and suppliers—that contribute to the in-store food environment, more healthy food marketing, and consumer food choice. Further, our study provides novel contextual information about supermarket managers’ decision-making processes and industry relationships. Additional research is needed to examine retailers’ perspectives at all levels of food retail (i.e., small or corner stores, convenience stores, small and large chain supermarkets, etc.) as well as in different demographic and geographic locations, in order to identify the most efficacious ways to intervene. As well, it would be important to seek perspectives from suppliers and distributors, and from individuals involved in more healthy food retail programming, to better understand their relationships with retailers. This can further delineate external forces influencing retailers’ food merchandising decisions and the parameters in which these decisions are made. Understanding factors that affect retailers’ in-store decision making provides important insights into how to improve the food environment and will facilitate development of effective and more long-term healthy food retail policy approaches and interventions.

## Abbreviations

ATLAS.ti: qualitative data analysis software
CDFI: Community Development Financial Institution
Four Ps: Product, placement, promotion and pricing retail strategies
GMO: Genetically modified organism
NYC: New York City
NYC DOHMH: New York City Department of Health and Mental Hygiene
NYS: New York State
RWJF: Robert Wood Johnson Foundation
